# Parasite Load Induces Progressive Spleen Architecture Breakage and Impairs Cytokine mRNA Expression in *Leishmania infantum*-Naturally Infected Dogs

**DOI:** 10.1371/journal.pone.0123009

**Published:** 2015-04-13

**Authors:** Amanda S. Cavalcanti, Marcelo Ribeiro-Alves, Luiza de O. R. Pereira, Gustavo Leandro Mestre, Anna Beatriz Robottom Ferreira, Fernanda N. Morgado, Mariana C. Boité, Elisa Cupolillo, Milton O. Moraes, Renato Porrozzi

**Affiliations:** 1 Laboratório de Pesquisas em Leishmaniose, Instituto Oswaldo Cruz, Fiocruz, Rio de Janeiro, Brasil; 2 Laboratório de Pesquisa Clínica em DST-AIDS, Instituto de Pesquisa Clínica Evandro Chagas, Fiocruz, Rio de Janeiro, Brasil; 3 Laboratório Interdisciplinar de Pesquisas Médicas, Instituto Oswaldo Cruz, Fiocruz, Rio de Janeiro, Brasil; 4 Centro de Controle de Zoonoses, Secretaria Municipal de Saúde, Cuiabá, Brasil; 5 Laboratório de Hanseníase, Instituto Oswaldo Cruz, Fiocruz, Rio de Janeiro, Brasil; INRS - Institut Armand Frappier, CANADA

## Abstract

Canine Visceral Leishmaniasis (CVL) shares many aspects with the human disease and dogs are considered the main urban reservoir of *L*. *infantum* in zoonotic VL. Infected dogs develop progressive disease with a large clinical spectrum. A complex balance between the parasite and the genetic/immunological background of the host are decisive for infection evolution and clinical outcome. This study comprised 92 Leishmania infected mongrel dogs of various ages from Mato Grosso, Brazil. Spleen samples were collected for determining parasite load, humoral response, cytokine mRNA expression and histopathology alterations. By real-time PCR for the ssrRNA Leishmania gene, two groups were defined; a low (lowP, n = 46) and a high parasite load groups (highP, n = 42). When comparing these groups, results show variable individual humoral immune response with higher specific IgG production in infected animals but with a notable difference in CVL rapid test optical densities (DPP) between highP and lowP groups. Splenic architecture disruption was characterized by disorganization of white pulp, more evident in animals with high parasitism. All cytokine transcripts in spleen were less expressed in highP than lowP groups with a large heterogeneous variation in response. Individual correlation analysis between cytokine expression and parasite load revealed a negative correlation for both pro-inflammatory cytokines: IFNγ, IL-12, IL-6; and anti-inflammatory cytokines: IL-10 and TGFβ. TNF showed the best negative correlation (r^2^ = 0.231; p<0.001). Herein we describe impairment on mRNA cytokine expression in leishmania infected dogs with high parasite load associated with a structural modification in the splenic lymphoid micro-architecture. We also discuss the possible mechanism responsible for the uncontrolled parasite growth and clinical outcome.

## Introduction

Canine Visceral Leishmaniasis (CVL) shares many aspects with the human disease and dogs are considered the main urban reservoir of *L*. *infantum* in zoonotic VL. Canine infection may precede the emergence of human cases [1] and the presence of infected dogs is directly associated with the risk of human infection [2]. The control programs of VL in endemic areas of Latin America include the detection and treatment of infected and sick humans, insecticide spraying in residential outhouses and selective removal of seropositive dogs. Screening and mass culling of seropositive dogs has not been proved to be uniformly effective in control programs [3] and many studies have questioned its effectiveness [4–7]. Therefore, the knowledge of the immune mechanisms involved in animal pathology and protection plays a pivotal role in the endemic control [8].

Infected dogs develop progressive disease, characterized by lymphadenopathy, hepatosplenomegaly, onychogryphosis, body weight loss, dermatitis, anemia and ultimately death. The large spectrum of clinical presentations ranges from asymptomatic to symptomatic infection [9]. A complex balance between the parasite and the genetic/immunological background of the host are decisive for the progression towards disease. However, no conclusive data are available on the immunological mechanisms responsible for resistance or disease progression in CVL. The infection is characterized by a marked humoral response [10,11] and the parasite load follows the clinical outcome [12]. Several studies show a mixed cellular response related to infection [2,13–15]. Such a mixed response is also observed under different experimental conditions [16]. The immune response to viscerotropic *Leishmania* parasites is organ-specific [17–19] and the spleen is an important target in VL [20]. Overall, in spleen the production of Th1 cytokines (such as IFN-γ, IL-12 and TNF) of both asymptomatic and symptomatic dogs does not show any differences [13,14,20], however they are increased during infection [14]. The predominance of Th2/regulatory cytokines (such as IL-4, IL-10 and TGF-β1) determines the parasite load and persistence without association with clinical groups [14,15]. Nevertheless, Correa et al. [13] found that these cytokines are determinant for disease progression. This organ is a site of parasite persistence where the parasites grow slowly generating important changes both in architecture and organ function. Also, a relationship between a high percentage of T cell apoptosis and the structural disorganization of white pulp may co-contribute to the inefficient cellular-mediated-immune response in CVL [21].

Herein we describe impairment in cytokine mRNA expression in naturally *Leishmania infantum* infected dogs with high parasite load associated with a structural modification of the lymphoid micro-architecture in spleen. We also discuss the possible mechanism responsible for the uncontrolled parasite growth and clinical outcome.

## Methods

### Ethics Statement

The infected animals included in this study were destined to euthanasia as recommended by the politics of Brazilian Ministry of Health at the Center for Zoonosis Control (CZC). The study has been conducted in accordance to AVMA Guidelines for the Euthanasia of Animals [22]. For euthanasia, dogs were anesthetized with an intravenous injection of 1.0% (1.0 ml/kg) thiopental (Thiopentax, Cristália). Once the absence of corneal reflex induced by deep anesthesia was observed, 10.0 mL of 19.1% Potassium Chloride (Isofarma) were administered by intravenous injection. The Animal Care and Use Committee of Fundação Oswaldo Cruz does not require ethical clearance in these cases, since the animal were not submitted to any experimental procedure. The samples were collected for diagnostics purposes. Informed consent was obtained from all dog’s owners.

### Study Animals and Clinical Evaluation

The study comprised 92 IFAT-positive mongrel dogs of various ages, with anti-Leishmania IgG antibody titers higher than 1:40. The infected animals were destined to euthanasia following owner consent at the Center for Zoonosis Control (CZC) of four endemic municipalities (Rondonópolis, Barra do Garças, Várzea Grande and Cuiabá) in Mato Grosso, Brazil. Infection was confirmed in all IFAT-positive dogs by one additional serological test, being either ELISA, rapid test Dual Path Platform (DPP CVL, BioManguinhos, FIOCRUZ) and/or parasite detection by culture and/or conventional PCR (kDNA). The infection etiology was confirmed by MLEE in all isolated strains at the *Leishmania* Collection of the Oswaldo Cruz Institute (CLIOC, www.clioc.fiocruz.br). Isolated strains were deposited as open access. Serum samples from noninfected dogs from a nonendemic area, Rio de Janeiro, RJ, Brazil (control group, n = 15) were also included in serologic analyses. Clinical evaluation was performed by two veterinarians according to the clinical scale adapted from Quinnel and co-workers [23]. In summary, six common signs (dermatitis, onycogryphosis, conjunctivitis, emaciation, alopecia and lymphadenopathy) were scored on a semiquantitative scale from 0 (absent) to 3 (severe). The sum of values was used to achieve the final clinical classification as low (0–2), medium (3–6) or high (7–18) score.

### Sample collection and storage

Blood samples were collected from the cephalic vein and serum was stored at -20°C. Immediately after euthanasia, fragments of spleen were harvested and stored in net buffer solution (10 mM NaCl, 10 mM EDTA, 10 mM Tris HCl) for DNA extraction and in an RNAlater Tissue Collection solution (Ambion, Applied Biosystems, Life Technologies Corporation) for RNA extraction. The biopsies were frozen and stored at -70°C prior to processing. Tissue fragments were fixed in buffered formalin for histology. Needle aspirate was seeded in NNN-Schneider *Drosophila* (Sigma-Aldrich) for parasite isolation.

### Serology

The enzyme immunoassay with EIE—Leishmaniose Visceral Canina kit (BioManguinhos, FIOCRUZ) was performed according to the manufacturer with minor modifications. Briefly, sensitized microplates were incubated with diluted dog sera (1:10) at room temperature for 2 hours. Plates were incubated at room temperature for 1 hour with 100 μl of IgG (1:3000, Bethyl Laboratories) and IgM (1:1000, Bethyl Laboratories). The lower limit of positivity (cutoff) was determined by using the mean plus 3 standard deviations of the controls. Sera with OD values equal to or greater than cutoff value were considered positive and OD values below cutoff value considered negative. DPP-CVL was performed as instructed and read in a rapid test reader.

### Determination of Parasite burden by Quantitative Polimerase Chain Reaction (qPCR)

Total DNA was extracted from approximately 30 mg of spleen samples. DNA extraction was carried out by the Wizard Genomic DNA Purification System (Promega, Madison, WI, USA) which included a prior digestion phase with 17.5 μl of proteinase K (20 mg/mL) for 12 h at 55°C. DNA was dissolved in 100 μl of tris EDTA buffer (TE buffer). Parasite burdens were estimated by qPCR in spleen samples amplifying small subunit ribosomal RNA (ssrRNA, multy-copy gene) using primers described by Prina et al.[24], while HPRT primers were used to normalize concentrations of canine DNA in each sample (S1 Table). The qPCR reactions were run on the Step One equipment using Power Sybr Green Master Mix (Applied Biosystems, Molecular Probes, Inc.). Purified total DNA (100 ng) were added to a final PCR reaction volume of 20 μl containing Power Sybr Green 1X (Applied Biosystems, Molecular Probes, Inc.), 300 nM of each primer for HPRT or 500 nM for ssrRNA PCR assays. qPCR was performed with an activation step at 95°C for 10 minutes, followed by 40 cycles of denaturation, annealing/extension and reading (95°C for 15 seconds, 60°C for 1 minute and 68°C for 30 seconds) in a Step One termocycler (Applied Biosystems). A melt curve stage was performed for each specific amplification analysis (95°C for 15 seconds, 60°C for 1 minute and 95°C for 15 seconds). All reactions were performed in duplicate for each target and both targets were run on the same plate for the same sample.

Standard curves for HPRT and ssrRNA genes were prepared using serial 10-fold dilutions from 10^–2^ to 10^7^ of total purified DNA extracted wither from *L*. *infantum* (1x10^6^) or peripheral blood mononuclear cells (PBMCs). A threshold of detection was set for each target gene according to the background level from cycles 6–15 of all valid reactions. Mean threshold cycle (Ct) values were determined for technical duplicates. Ct values were plotted against input log-dilutions (base 10) and standard curves for each target determined by a linear regression, with the determined coefficients of determination (R2) used as quality control. Subsequently, the fitted standard curves were used to estimate overall number of parasites in the sample, while the host HPRT gene was used for PBMC number normalization. Thus, it was possible to obtain the number of parasites per 10^6^ cells. Amplification efficiency of each target was determined according to the equation: E = 10^(-1/slope). Data processing and presentation were performed using routines written in the R language, for the R statistical package version 2.922 [25].

### Parasite burden group determination

The number of groups was defined by the parasite burden, using the estimated log number of parasites per 10^6^ cells (base = 10) for each sample. The parasite number cut-off that delimited both groups was optimized by the fitting of mixtures of normal distributions by the standard expectation-maximization (EM) algorithm combined with a non-parametric likelihood ratio statistics with 1,000 permutations for testing the null hypothesis of a k-component fit versus the alternative hypothesis of a (k+1)-component fit to various mixture models, up to a specified number of maximum components, k (k = 5) [26]. A p-value was calculated for each test and once the p-value was above the significance level of 0.05, the test was terminated. These analyses were performed using the mixtools library for the R statistical package version 2.922 [25].

### Cytokine gene expression

Total RNA from 50–100 mg of tissue samples was isolated using Trizol Reagent (Invitrogen, Grand Island, NY), according to the manufacturer's protocol. DNase treatment to digest genomic DNA that could lead to false positive gene expression results was accomplished using DNA-free DNase (Ambion, Grand Island, NY). RNA integrity was confirmed on a 3-(N-morpholino) propanesulfonic acid/formamide 1.2% agarose gel stained with SYBR Nucleic Acid Gel Stain (Molecular Probes, Invitrogen Corp., Grand Island, New York). RNA quantity was assessed using a Nanodrop spectrophotometer (Thermo Scientific, Waltham, MA). For cDNA synthesis, 1.0 μg of RNA was reverse transcribed with oligo (d)T primers using the ImProm-II Reverse Transcription System (Promega, Madison, WI), according to the manufacturer's protocol including ribonuclease inhibitor (Recombinant RNasin, Promega, Madison, WI). Reverse transcription reactions were performed in duplicate at a final volume of 20 μL and diluted (1:4) by adding 80 μL of nuclease free water. Reactions without the reverse transcriptase enzyme (No-RT reactions) were performed to control DNA contamination. The qPCR reactions were run at a final volume of 20 μL containing 300 nM of primers, 1X SYBR GREEN master mix (Applied Biosystems) and 4 μL of cDNA template. qPCR was performed with an activation step at 95°C for 10 minutes, followed by 40 cycles of denaturation and annealing/extension (95°C for 10 seconds and 58°C for 1 minute). A melt curve stage was performed for each specific amplification analysis (95°C for 15 seconds, 60°C for 1 minute and 95°C for 15 seconds). All reactions were performed in triplicate in a Step One Plus termocycler (Applied Biosystems).

### Gene expression analysis of qPCR data

The fluorescence accumulation data from triplicate qPCR reactions for each sample were used to fit four-parameter sigmoid curves to represent each amplification curve using the qPCR library [27] for the R statistical package version 2.922 [25]. A detailed description of quantitation using Cp (crossing point) can be obtained elsewhere [28]. Genes used in the normalization between the different amplified samples were selected by the geNorm method [29] among a set housekeeping genes (S1 Table). The comparison of means of normalized gene expression values among groups were performed by: (1) a nonparametric T-test with 1,000 permutations for two groups; (2) a nonparametric one-way ANOVA with 1,000 unrestricted permutations, followed by pair-wise comparisons with Bonferroni adjustment, for more than 2 groups. Results were represented in graphs displaying the expression level mean ± standard error of mean for each group. Two-tailed levels of significance less than or equal to 0.01, 0.05 and 0.1 were considered "highly significant", "significant" and “suggestive”, respectively. Relationships between differentially expressed gene and sample profiles was investigated by Bayesian infinite mixtures model cluster analysis [30] and represented by 2D heatmaps and dendograms.

### Histopathology

Spleen fragments were fixed in 10% buffered formalin, embedded in paraffin and sliced in 5-μm thick sections mounted on microscope slides. The sections were stained with haematoxylin and eosin and examined by light microscopy (Nikon Eclipse E400—Tokyo, Japan). Structural changes of spleen lymphoid tissue, cell population in the red pulp and parasite burden were analyzed as described by Santana et al [31]. Briefly, the parameters analyzed included perisplenitis (absent, low, average or high), presence of granuloma and degree of white pulp structural organization (1-well organized: with distinct periarteriolar lymphocyte sheath, germinal center, mantle zone and marginal zone; 2-slightly disorganized: with either hyperplastic or hipoplastic changes leading to a loss in definition of any of the regions of the white pulp; 3-moderately disorganized: when the white pulp was evident, but its regions were poorly individualized or indistinct; and, 4-extensively disorganized: when the follicular structure was barely distinct from the red pulp and T-cell areas). The frequency of lymphoblasts, macrophages, neutrophils and plasma cells in the red pulp were scored as low, average or high. The amount of amastigotes was estimated by counting 40 to 100 fields (x1000 magnification) per section equally distributed between the sub-capsular compartment and the internal red pulp. The results were expressed as ratio of fields with amastigotes/total fields evaluated.

## Results

### Clinical characteristics and spleen parasite load

**Fig 1:**
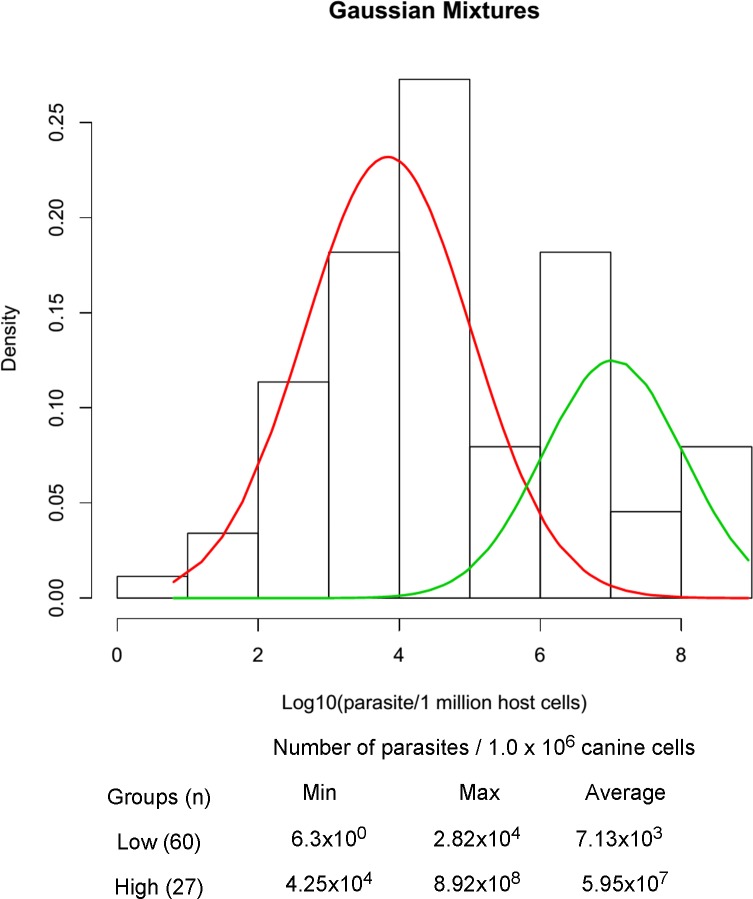
Quantification of parasite load in spleen samples from dogs naturally infected with *Leishmania infantum*. Quantification was carried out using real time PCR with primers specific for a *Leishmania* sp. DNA sequence of the small subunit ribosomal RNA (ssrRNA). The canine HPRT gene was used in order to normalize initial concentrations of DNA in each sample. (A) Histogram and normal density lines of *L*. *infantum* infected animals classified into low (n = 46), red line, or high (n = 42), green line, parasite load by the fitting of an optimum number of mixtures of normal distributions. (B) Minimum and maximum values of parasite per 10^6^ canine cells. Each sample was quantified in duplicate for each target. a,b Statistically significant differences (p < 0.0001, Unpaired T-test).

**Fig 2:**
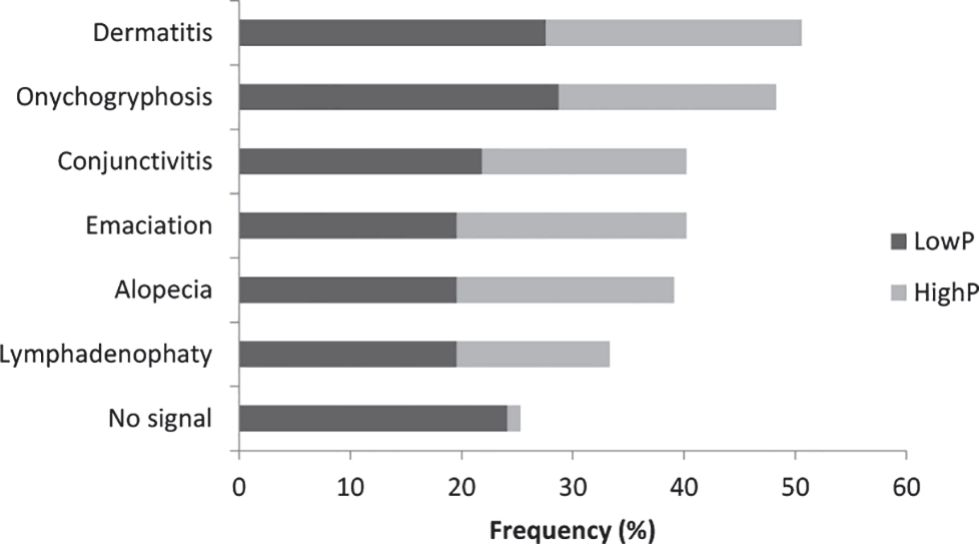
Most animals with high parasite load present clinical signs. Clinical signs in *Leishmania infantum* infected animals classified into low (n = 46) or high (n = 42) parasite DNA load measured by real-time PCR for the *Leishmania* ssrRNA gene in spleen.

Clinical evaluation was performed in 88 dogs according to the severity of signs where 33 animals were low, 29 medium and 26 high scored. All animals included in this study showed at least one positive parasitological test, including parasite culture and/or kDNA PCR. Spleen parasite load was determined by real-time PCR for the ssrRNA Leishmania gene and the group of animals with the least clinical score presented less parasite load (p = 0.01), but with a large variation (S1 Fig). Considering that clinical signs could be a result of uncontrolled factors such as coinfection, nutritional status and other disorders, a statistical analysis considering parasite load was performed resulting in the definition of two groups. Low parasite load group (lowP, n = 46) ranging from 6.3 x 10^0^ to 2.82 x 10^4^ and high parasite load group (highP, n = 42) ranging from 4.25 x 10^4^ to 8.92 x 10^8^ *Leishmania* genomes (Fig 1). The clinical signs observed were dermatitis (27.3 and 22.7%), onycogryphosis (28.4 and 19.3%), conjunctivitis (21.6 and 18.2%), emaciation (19.3 and 20.5%), alopecia (19.3 and 19.3%) and lymphadenopathy (19.3 and 13.6%) in the lowP and highP groups, respectively. Twenty-two animals (25.0%) did not show any clinical sign of leishmaniasis, of these 21 (23.9%) in the lowP and 1 (1.1%) in the highP group (Fig 2).

### Humoral Immune Response

**Fig 3:**
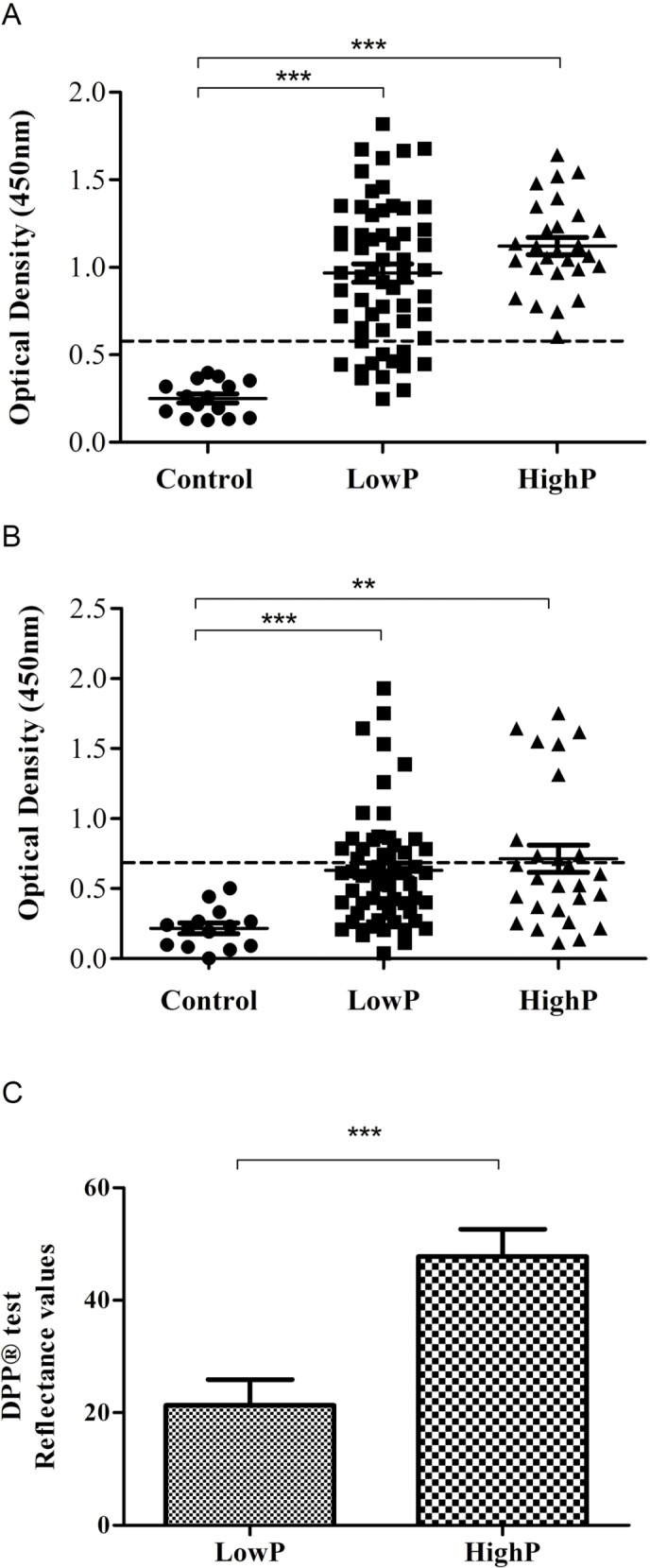
*Leishmania infantum* naturally infected dogs present increased levels of immunoglobulins. *Leishmania*-specific total IgG (A and C) and total IgM (B) levels in sera from noninfected dogs (control, n = 15) and from naturally infected dogs with either low (lowP, n = 46) or high (highP, n = 42) parasite load in spleen. Optical density of serum samples were determined by ELISA (A and B) using crude protein derived from *L*. *infantum* promastigotes IOC/L3128 (MCAN/BR/2009/BOB-FÍGADO). Reflectance values were determined by the rapid immunocromatographic test (Dual Path Platform, DPP, BioManguinhos). Horizontal bars indicate mean values. The dashed line indicates the cut-off, 0.549 for IgG and 0.645 for IgM. (***) p < 0.001; (**) p < 0.0002 indicate statistically significant differences, Mann Whitney test.

The production of anti-Leishmania IgG and IgM antibodies were evaluated in 15 uninfected dogs from a non-endemic area (control) and 86 dogs naturally infected by *L*. *infantum*, of which 59 belonged to the lowP and 27 to the highP group. The serum samples of two animals were lost during transport. The mean titers of anti-Leishmania IgG antibodies in the lowP group (OD 0.966 ± SD 0.403) and highP group (OD 1.121 ± SD 0.257) were similar, as well as IgM titers OD 0.630 ± SD 0.407 and OD 0.713 ± SD 0.507, for lowP and highP, respectively. In contrast, no positive serum reactivity occurred against the antigen in 15 control dogs for IgG (OD 0.251 ± SD 0.098) or IgM (OD 0.216 ± SD 0.143). Our results show variable individual humoral immune response with higher specific IgG production in infected animals compared to the control group, p < 0.0001 (Fig 3A). No statically significant difference was found between lowP and highP groups but there was an increased frequency of positivity in highP. IgM levels also increased with infection and it seems to be maintained along the infection since no differences were observed among infected groups (Fig 3B). Notably, the difference in the optical densities in DPP (rapid test) between highP and lowP groups was significant, p < 0.0001 (Fig 3C).

### Splenic cytokine gene expression

**Fig 4:**
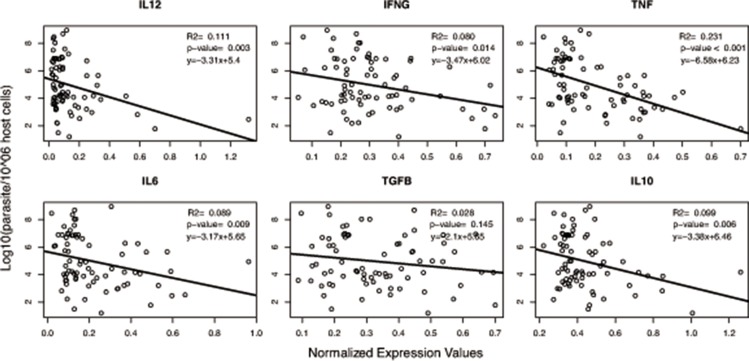
Pro-inflammatory and regulatory/anti-inflammatory cytokine mRNA expression correlates with low parasite load. Ex-vivo analyses of relative mRNA levels for indicated genes in the splenic compartments of mongrel dogs infected with *Leishmania infantum* were correlated with individual log of spleen parasite load detected by pPCR for the *Leishmania* sp ssrRNA gene. Gene expression levels of each tested cytokine were normalized using HPRT and RP32 expression. For parasite load values, canine HPRT primers were used in order to normalize initial concentrations of DNA in each sample. P value < 0.05 indicates significant correlation, Pearson correlation test.

**Fig 5:**
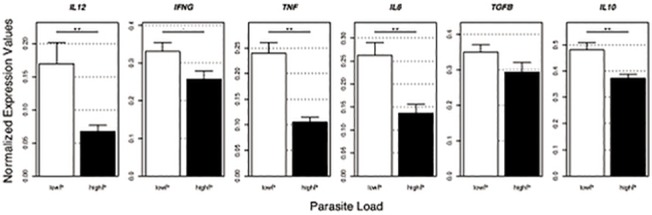
Splenic mRNA cytokines are down regulated in *Leishmania infantum* infected dogs with higher parasite loads. Ex-vivo analyses of relative mRNA levels for indicated genes in the splenic compartments of mongrel dogs infected with *L*. *infantum* and classified as low (lowP) or high (highP) parasite load. Gene expression levels of each tested cytokine were normalized using HPRT and RP32 expression. Error bars indicate the standard error of mean for each group. Significant differences are indicated by, p < 0.05 and **, p < 0.01 (nonparametric T-test with 1,000 permutations).

We first compared cytokine gene expression according to clinical groups and, besides TNF (p < 0.05) (S2 Fig), no other cytokine showed any differences. A large variation among groups was observed (S3 Fig). However, individual correlation analysis between cytokine expression and parasite load revealed none or very weak negative correlations for both pro-inflammatory cytokines: IFNγ (r = 0.080; p = 0.014), IL-12 (r = 0.111; p = 0.003), IL-6 (r = 0.089; p = 0.009); and anti-inflammatory cytokines: IL-10 (r = 0.099; p = 0.006), TGFβ (r = 0.028; p = 0.145). Among the cytokines, TNF showed the highest, although still very weak, negative correlation (r = 0.231; p < 0.001) (Fig 4). Since the primary interest is to determine the response to *L*. *infantum* infection, cytokine expression was analyzed according to parasite load groups (Fig 5). Cytokine transcripts in spleen were lesser expressed in highP than lowP groups. A heterogeneous, although significant variation in response among groups was obtained for IL-12 (0.169 ± 0.224 and 0.067 ± 0.048; p = 0.007), IFNγ (0.330 ± 0.165 and 0.257 ± 0.113; p = 0.059), TNF (0.239 ± 0.145 and 0.105 ± 0.048; p < 0.001), IL-6 (0.262 ± 0.193 and 0.136 ± 0.096; p < 0.001) and IL-10 (0.480 ± 0.198 and 0.372 ± 0.081; p = 0.003) in lowP and highP groups, respectively. There were no significant differences between groups in gene expression of IL-27 (0.009 ± 0.033 and 0.013 ± 0.047, p = 0.358, data not shown) and TGFβ (0.348 ± 0.154 and 0.293 ± 0.140; p = 0.149).

### Histopathology of splenic tissue

**Fig 6:**
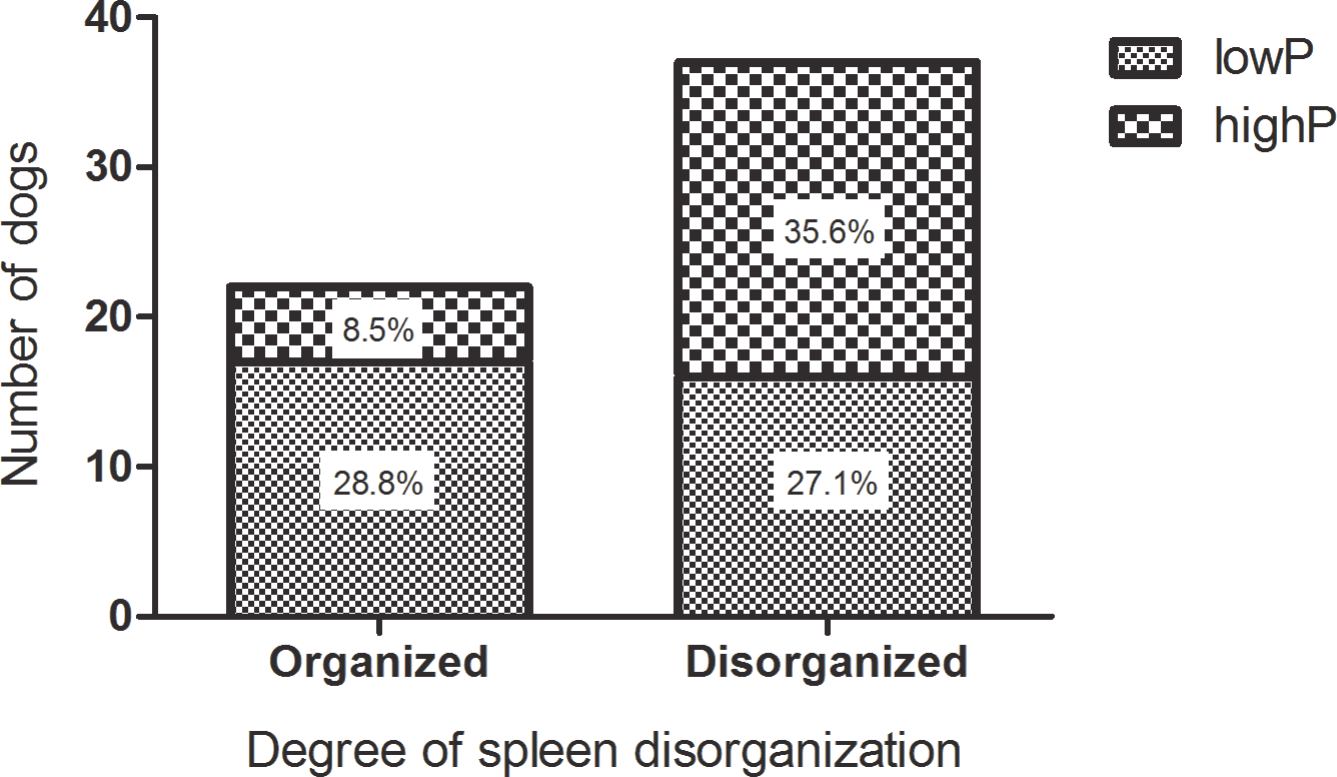
Dogs with high parasite load show moderately to extensively disorganized white pulp. Degree of white pulp organization by histopathology in *Leishmania infantum* infected animals classified into low (n = 33) or high (n = 26) parasite DNA load measured by real-time PCR for ssrRNA Leishmania gene in spleen.

**Fig 7:**
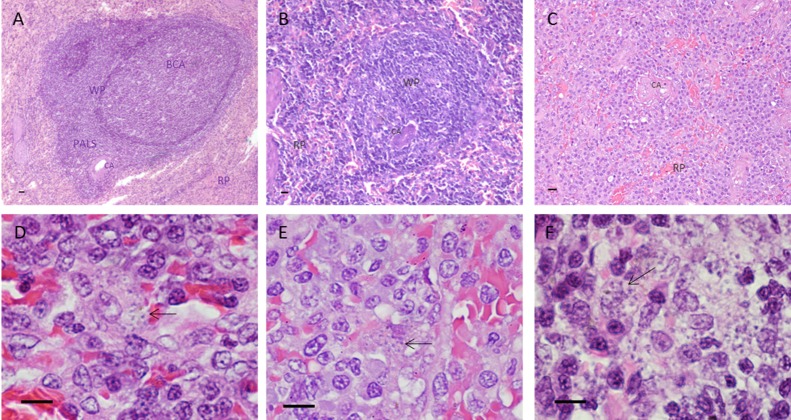
Morphological changes in the spleen of dogs naturally infected by *Leishmania infantum*. (A) Organized splenic white pulp compartments with well-formed follicle; (B) Moderately disorganized splenic white pulp when the white pulp was evident, however, its regions were poorly individualized or indistinct; (C) Disorganized splenic white pulp without compartment distinction; (D, E and F) Intracelullar amastigotes (arrows). HE, Scale bar 20 μm (A, B and C); 10 μm (D, E and F). WP—white pulp; RP—red pulp; BCA—B cell area; PALS—periarterial lymphoid sheat; CA—central artery.

The tissue organization was accessed in 67% (59/88) of the animals and the potential role of the parasite was analyzed. Overall, intense inflammation (perisplenitis), presence of granulomas in different stages of maturation, lymphoblasts, plasma cells, neutrophils and macrophages were observed (Table 1). Splenic architecture disruption was characterized by disorganization of white pulp, where in 22 out of 59 animals presented well organized splenic white pulp with 17 (28.8%) of those classified as lowP and only 5 (8.5%) as highP. On the other hand, 37 out of 59 showed moderately to extensive disorganized white pulp with 16 (27.1%) and 21 (35.6%) animals in lowP and highP group, respectively (p = 0.015; odds ratio = 4.463, Fisher's exact test) (Table 1 and Fig 6). Noteworthy, the direct observation of the number of amastigotes in the subcapsular compartment and in the red pulp corroborated the parasite load obtained by qPCR (Table 1 and Fig 7).

**Table 1:** Spleen histopathology of dogs naturally infected by *Leishmania infantum* (**) p < 0.001; (*) p < 0.01.

		Parasite Load	
Parameters		Low	High
**Number of animals**		33 (55.9%)	26 (44.1%)
**Positive fields of subcapsular amastigotes		10.2%	28.1%
**Positive fields of red pulp amastigotes		10.1%	26.1%
White Pulp*			
*well organized to slightly disorganized*		17 (28.8)	5 (8.5)
*moderately disorganized to extensively disorganized*		16 (27.1)	21 (35.6)
**Perisplenitis**	*absent—low*	24 (40.7)	18 (30.5)
	*average—high*	8 (13.6)	8 (13.6)
**Granuloma**	*absent*	26 (44.1)	16 (27.1)
	*presence*	7 (11.7)	10 (16.9)
**Lymphoblasts**	*absent—low*	10 (16.9)	4 (6.8)
	*average—high*	23 (39.0)	22 (37.3)
**Plasma Cells**	*absent—low*	13 (22.0)	5 (8.50)
	*average—high*	20 (34.0)	21 (36.0)
**Neutrophils**	*absent—low*	20 (34.0)	14 (23.7)
	*average—high*	13 (22.0)	12 (20.3)
**Macrophages**	*absent—low*	12 (20.3)	16 (27.1)
	*average—high*	21 (36.0)	10 (17.0)

Fisher's exact test.

## Discussion

Is widely known that *L*. *infantum*-infected dogs present a wide interindividual range of clinical signs yet our data indicate that no conclusive pattern of splenic immune response could be associated with clinical presentation. Herein we show that the intensity of tissue parasite seems to be determinant for immune response modulation. Applying a semi-quantitative arbitrary scale we first divided the animals by clinical score: low, medium or high. This division was not able to demonstrate statically significant differences neither for antibody response nor for parasite load. Except by TNF in low score group, no other cytokine evaluated revealed significant differences among clinical groups (S4 Fig). Other studies have also associated Th1 cytokine expression, including TNF, with asymptomatic infection [32,33]. Although the magnitude of cytokine expression varied markedly, parasite load revealed a negative correlation for all assayed cytokines (Fig 4), unlike reported by other authors [34]. In an effort to evaluate the role of the parasite in the spleen, the animals were split in two groups (lowP and highP) by statistical analysis according to splenic *Leishmania* DNA load in spleen.

Serology by ELISA for *Leishmania* specific IgG or IgM demonstrated no significant difference between highP and lowP groups and an extensive range of reactivity was observed. However, we found a higher frequency of reactivity in the highP than lowP group for IgG and the opposite for IgM. It might indicate that lowP might represent more recently infected animals. This result was corroborated by response detected through DPP test. Although it can be useful to confirm clinically suspected cases, the DPP CVL rapid test is not sensitive enough for detecting asymptomatic canine carriers of *L*. *infantum* [35]. We demonstrate that the reflectance values in the DPP test can be related to parasite load. In light of leishmaniasis control, further studies are needed to confirm if values in the DPP test could be related with the potential for dogs to transmit parasites.

The splenic effector response was assayed by RT-qPCR used for mRNA levels detection of both pro-(IFNγ, IL-12, TNF and IL-6) and anti-inflammatory/regulatory (TGFβ and IL-10) cytokines. The correlation between parasite load and mRNA cytokine expression indicates that despite of the remarkable variation in terms of expression, all of them were reduced in heavily infected animals and this could suggest, at some level, immunosupression. Notably, even in experimental infection under controlled conditions, there is a high variable response, suggesting an individual modulation of the immune response by the host [16]. We also observed an association between increasing parasite load and micro-architecture rupture of the spleen. The alterations observed varied from a well-organized white pulp to an extensive structural disorganization. It consists of hyper or hipoplastic changes in the white pulp and changes in follicular structure. These various levels of splenic organization in the dogs were correlated with increased parasite load. Such a breakdown in tissue architecture related to VL has been previously reported in human [36–38], murine [39,40] and canine infections [31,41–43]. The development of splenic pathology is associated with disease progression in dogs [31]. In mice model of *L*. *donovani* infection, the splenic pathology was associated with high levels of TNF irrespective of parasite burden [44]. Of all the cytokines evaluated, TNF and IL-12 were more markedly reduced (2.3x and 2.5x respectively) with parasite load increase. Higher TNF mRNA levels in LowP group seems to control the parasite growth, but on the other hand can generate tissue damage. The role of TNF and IL-12 in VL has been described in the literature, both in experimental infection of mice [45–48] as in canine infection [15,20,49,50]. Both are pro-inflammatory cytokines involved in systemic inflammation that stimulate the acute phase reaction. Interleukin 12 is a multifunctional cytokine acting as a key regulator of cell-mediated immune responses through the differentiation of naïve CD4+ T cells into type 1 helper T cells (Th1) producing interferon-gamma (IFNγ) [51]. These cytokines play a pivotal role in the pathogenesis of many chronic autoimmune diseases [52–54] and are also crucial for the control of intracellular microorganisms [55,56]. The activation cellular immune responses is associated with IL-12/IFNγ axis that leads to intracellular killing of parasites. When IFNγ-treated cells are infected with pathogens, they are stimulated to make TNF [57]. Notably, VL is an opportunistic infection in patients under biological therapy with anti-TNF drugs [58]. TNF cellular responses can eradicate infectious agents, but can also lead to local tissue injury at sites of infection and harmful systemic effects [56].

Cytokine transcripts in spleen revealed a high heterogeneous response among groups and in the highP group pro-inflammatory cytokines (IFNγ, TNF, IL-12 and IL-6) showed a larger reduction in expression than anti-inflammatory/immunoregulatory (IL-10 and TGFβ) cytokines. These data suggest regulatory mechanisms to prevent tissue damage or increasing fibrosis leading to leakage of parasite control CVL. While clinical resistance has been shown to be associated with the predominant expression of Th1 cytokines, such as IL-2, IFNγ and TNF, susceptibility and parasite persistence is characterized by predominance of Th2 and immune-regulatory cytokines, such as IL-4 and IL-10 [59]. Nevertheless, our results, as well as several other studies, do not support such associations, showing a mixed response to infection [2,13–15]. There is no consensus about the immunological role for a functional T cell phenotype concerning cytokine production in spleen. On the other hand, CD8 T cell exhaustion and the PD-1 (programed cell deth—1) molecule function has been recently described in viral and parasitic infections [60,61]. Also, it was demonstrated T cell exhaustion in splenocytes of human patients with VL[62]. Recently, a PD-1–mediated pan-T cell exhaustion has been shown in peripheral blood of infected dogs with subsequent reduction in cytokine expression [63]. In this context, general reduction of cytokine expression could be also related to exhaustion induced by the excess of circulating antigen in animals with high parasite load.

In conclusion, this study demonstrated the rupture of splenic architecture and failure in cytokine mRNA expression in animals with high splenic parasitism during CVL. Inflammatory cytokine environment [15,16] and possibly proteolytic enzymes [64] produced early in infection cause the progressive destruction of the architecture with loss of marginal zone macrophages and stromal cells affecting cell migration, antigen presentation and lymphocyte activation, as observed in murine experimental infection [44,65]. Consequently, there is a widespread decline in pro-inflammatory cytokines, as we observed, and chemokine expression, as previously reported [34,42], with consequent loss of parasite control or even due to an excess of parasite antigens leading to a strong local and tissue specific immunosuppression.

## Supporting Information

S1 Fig Animals with lower clinical score presented a lower spleen parasite load. Mongrel dogs infected with *Leishmania infantum* were classified into low (n = 33), medium (n = 29) or high (n = 26) clinical score groups. Parasite DNA load was achieved by real-time PCR for ssrRNA Leishmania gene in spleen. Canine HPRT gene was used in order to normalize initial concentrations of DNA in each sample. The horizontal bars indicate mean values. (**) p < 0.01; (*) p < 0.05 indicate statistically significant differences, Mann Whitney test.
(JPG)

S2 Fig Splenic mRNA TNF is down regulated in *Leishmania infantum* infected dogs with higher clinical scores. Ex-vivo analyses of relative mRNA levels for indicated genes in the splenic compartments of mongrel dogs infected with *L*. *infantum* and classified into low (n = 33), medium (n = 29) or high (n = 26) clinical score groups. Gene expression levels of each tested cytokine were normalized using HPRT and RP32 expression. Error bars indicate the standard error of mean for each group. (^.^) p < 0.1, nonparametric one-way ANOVA with 1,000 unrestricted permutations, followed by pair-wise comparisons with Bonferroni adjustment.
(JPG)

S3 Fig Naturally infected animals present a high heterogeneous cytokine response independent of clinical presentation. Heat map of differentially expressed genes from animals in different clinical groups. Clinical score was accessed and animals were classified as low (0–2), medium (3–6) or high score (7–18). Red corresponds to higher gene expression levels.
(TIF)

S4 Fig Declining trend of splenic cytokines mRNA according to spleen organization in *Leishmania infantum* infected dogs. Ex-vivo analyses of relative mRNA levels for indicated genes in the splenic compartments of mongrel dogs infected with *L*. *infantum* are shown in animals with different degrees of white pulp organization by histopatology. Gene expression levels of each tested cytokine were normalized using HPRT and RP32 expression. Error bars indicate the standard error. Mann Whitney test.
(TIF)

S1 Table Target genes and primers. (DOCX)
