# Supplementary figures and images for: Parasite Load Induces Progressive Spleen Architecture Breakage and Impairs Cytokine mRNA Expression in Leishmania infantum-Naturally Infected Dogs
Source: PLoS One. 2015 Apr 13;10(4):e0123009. doi: 10.1371/journal.pone.0123009 (PMC4395300; doi:10.1371/journal.pone.0123009)

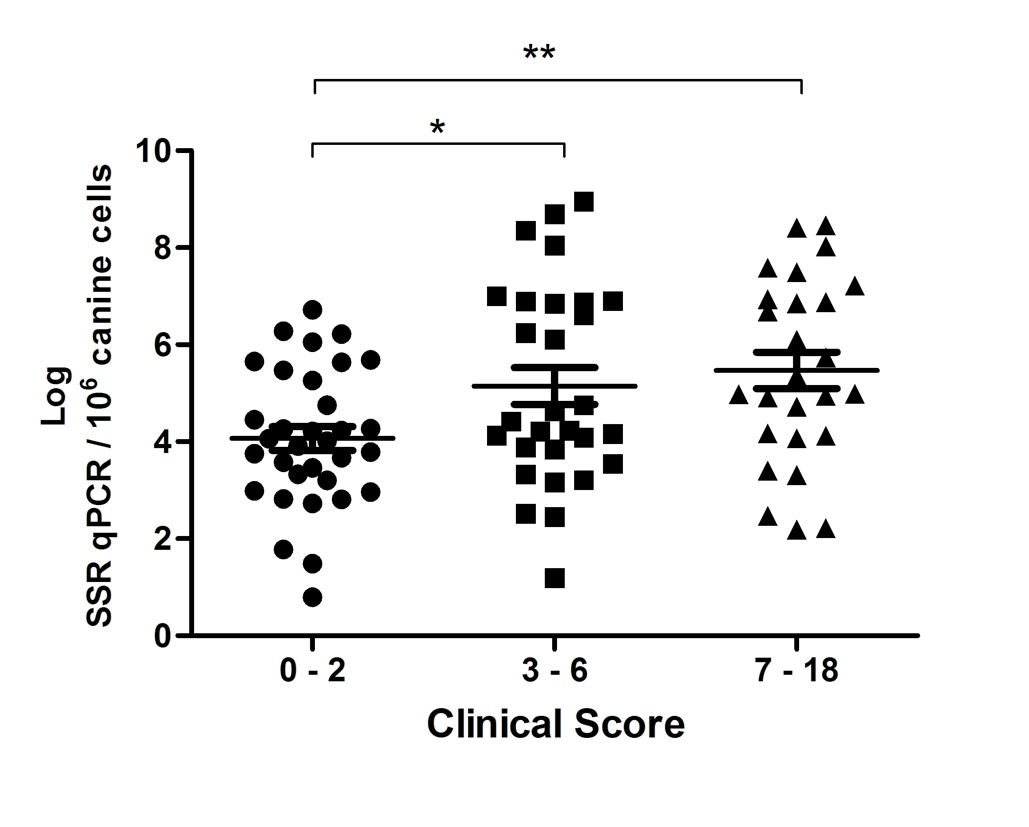

Supplement: S1 Fig — Mongrel dogs infected with Leishmania infantum were classified into low (n = 33), medium (n = 29) or high (n = 26) clinical score groups. Parasite DNA load was achieved by real-time PCR for ssrRNA Leishmania gene in spleen. Canine HPRT gene was used in order to normalize initial concentrations of DNA in each sample. The horizontal bars indicate mean values. (**) p < 0.01; (*) p < 0.05 indicate statistically significant differences, Mann Whitney test. (JPG) [file pone.0123009.s001.jpg]

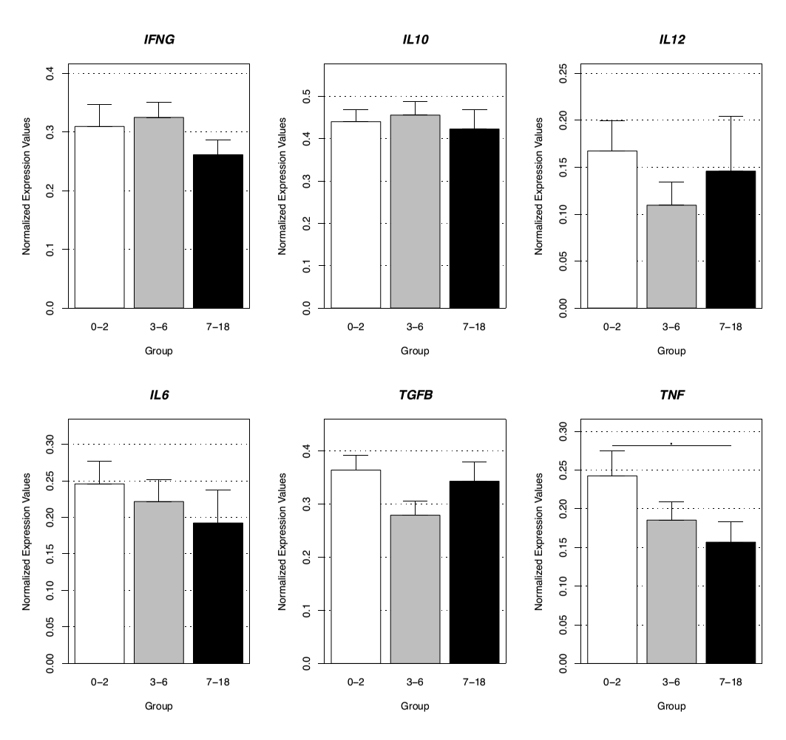

Supplement: S2 Fig — Ex-vivo analyses of relative mRNA levels for indicated genes in the splenic compartments of mongrel dogs infected with L. infantum and classified into low (n = 33), medium (n = 29) or high (n = 26) clinical score groups. Gene expression levels of each tested cytokine were normalized using HPRT and RP32 expression. Error bars indicate the standard error of mean for each group. (.) p < 0.1, nonparametric one-way ANOVA with 1,000 unrestricted permutations, followed by pair-wise comparisons with Bonferroni adjustment. (JPG) [file pone.0123009.s002.jpg]

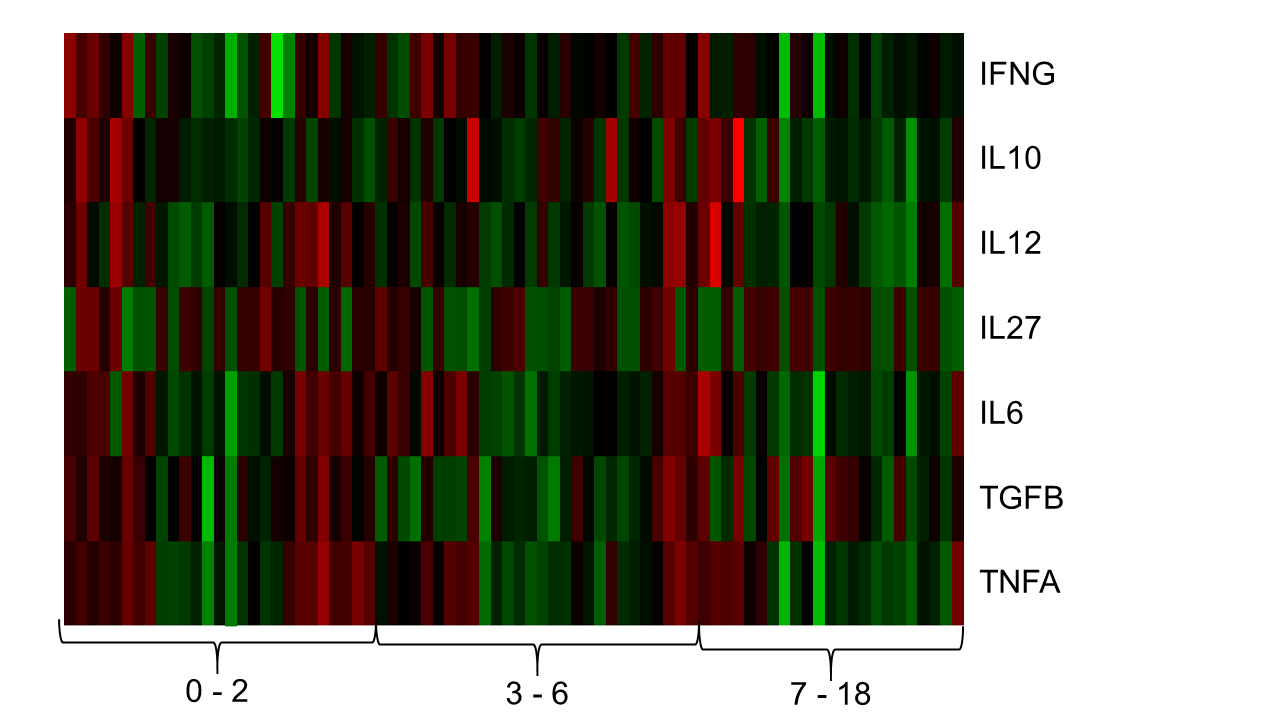

Supplement: S3 Fig — Heat map of differentially expressed genes from animals in different clinical groups. Clinical score was accessed and animals were classified as low (0–2), medium (3–6) or high score (7–18). Red corresponds to higher gene expression levels. (TIF) [file pone.0123009.s003.tif]

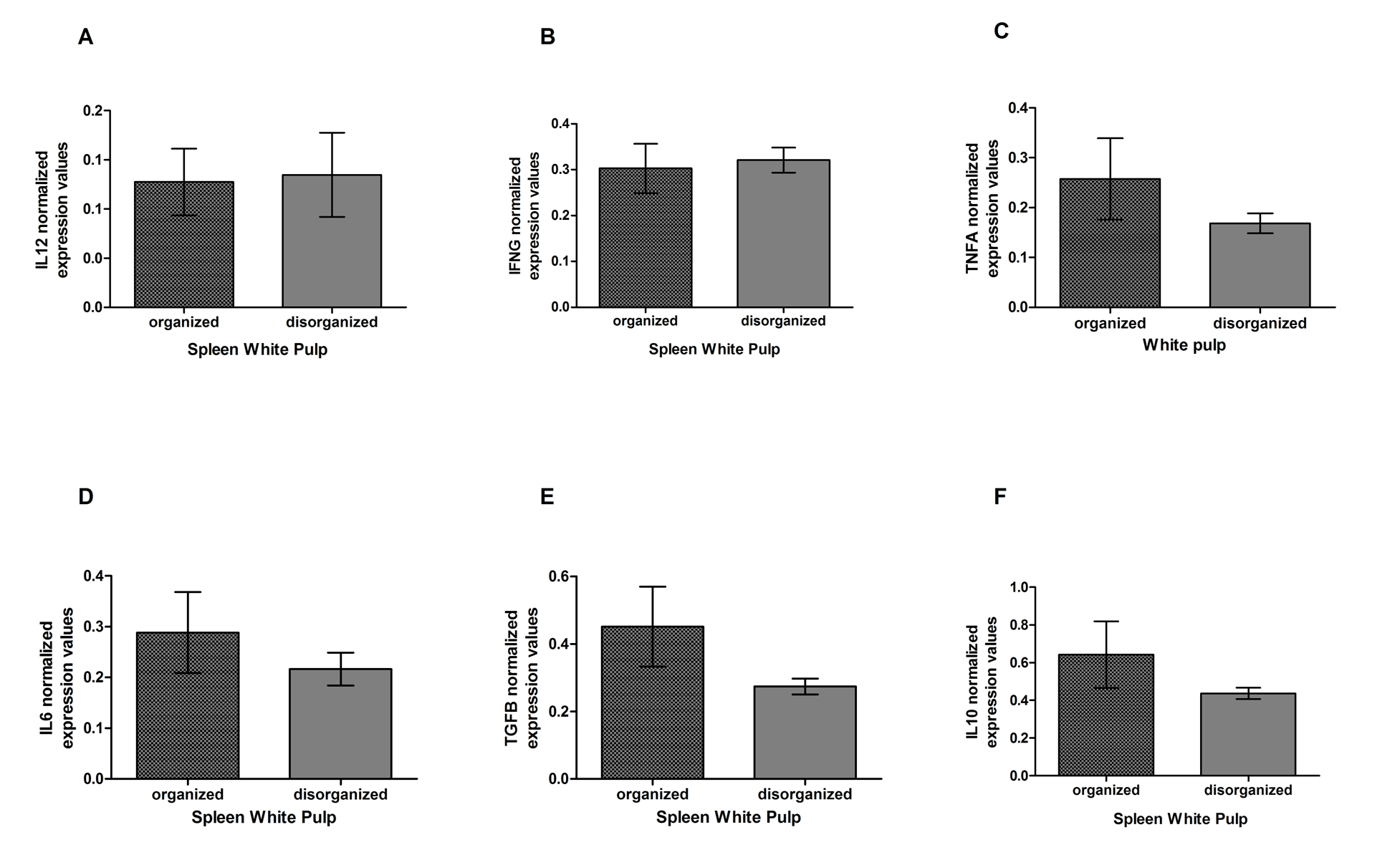

Supplement: S4 Fig — Ex-vivo analyses of relative mRNA levels for indicated genes in the splenic compartments of mongrel dogs infected with L. infantum are shown in animals with different degrees of white pulp organization by histopatology. Gene expression levels of each tested cytokine were normalized using HPRT and RP32 expression. Error bars indicate the standard error. Mann Whitney test. (TIF) [file pone.0123009.s004.tif]
