# Supplementary material for: Parasite Load Induces Progressive Spleen Architecture Breakage and Impairs Cytokine mRNA Expression in Leishmania infantum-Naturally Infected Dogs
Source: PLoS One. 2015 Apr 13;10(4):e0123009. doi: 10.1371/journal.pone.0123009 (PMC4395300; doi:10.1371/journal.pone.0123009)
Supplement: S1 Table — (DOCX) [file pone.0123009.s005.docx]

Table S1. Target genes and primers

| Target gene | Acession Number | Sequence (5’- 3’) | | Product (bp) | |
| --- | --- | --- | --- | --- | --- |
| IL-10 | NM_001003077.1 | forward | GAGAGAAGCTCAAGACCCTCAG | 118 |  |
|  |  | reverse | TGGAGCTTACTAAATGCGCTCT |  |  |
| TNF | NM_001003244.4 | forward | CAAATGGCCTCCAACTAATCA | 100 |  |
|  |  | reverse | TCGGGGTTTGCTACAACAT |  |  |
| IL-6 | NM_001003301.1 | forward | TCCAGAACAACTATGAGGGTGA | 100 |  |
|  |  | reverse | TCCTGATTCTTTACCTTGCTCTT |  |  |
| TGF-β | NM_0010033309.1 | forward | CTGGAGTCGTGAGGCAGTG | 96 |  |
|  |  | reverse | GCAGTGTGTTATCTTTGCTGTCA |  |  |
| IFN-γ | NM_001003174.1 | forward | CCAGATCATTCAAAGGAGCA | 116 |  |
|  |  | reverse | CGTTCACAGGAATTTGAATCAG |  |  |
| IL-12p40 | NM_001003292.1 | forward | TGGAGGTCAGCTGGGAATAC | 120 |  |
|  |  | reverse | CCACGCAGAGTATATCTTTCTTT |  |  |
| GADPH | XM_003434387.2 | forward | CCAGGTGGTCTCCTGTGACT | 103 |  |
|  |  | reverse | CCAGGAAATGAGCTTGACAAA |  |  |
| RP32 | NM_001252169.1 | forward | ATGCCCAACATTGGTTATGG | 181 |  |
|  |  | reverse | CTCTTTCCACGATGGCTTTG |  |  |
| HPRT  (cDNA) | NM_001003357.1 | forward | CCAGTCAACAGGGGACATAAA | 128 |  |
|  |  | reverse | TGACCAAGGAAAGCAAAGTC |  |  |
| HPRT  (DNA) | NW_003726126.1 | forward | AAAACAATGCAGACTTTGCT | 58 |  |
|  |  | reverse | CCTTGACCATCTTTGGATTA |  |  |
| *ssrRNA  (DNA) | AF303938;AY495829;M80293;M80295 | forward | TACTGGGGCGTCAGAG | 153 |  |
|  | M81416;M81422;M81427;M81429;M81430 | reverse | GGGTGTCATCGTTTGC |  |  |

*Prina et al. (2007)
